# Mitochondrial Function and Parkinson’s Disease: From the Perspective of the Electron Transport Chain

**DOI:** 10.3389/fnmol.2021.797833

**Published:** 2021-12-09

**Authors:** Jeng-Lin Li, Tai-Yi Lin, Po-Lin Chen, Ting-Ni Guo, Shu-Yi Huang, Chun-Hong Chen, Chin-Hsien Lin, Chih-Chiang Chan

**Affiliations:** ^1^Department of Neurology, National Taiwan University Hospital, Taipei, Taiwan; ^2^Division of Neurology, Department of Internal Medicine, Lo-Hsu Medical Foundation, Lotung Poh-Ai Hospital, Yilan County, Taiwan; ^3^College of Medicine, National Taiwan University, Taipei, Taiwan; ^4^National Institute of Infectious Diseases and Vaccinology, National Health Research Institutes, Miaoli County, Taiwan; ^5^Graduate Institute of Physiology, National Taiwan University, Taipei, Taiwan; ^6^Department of Medical Research, National Taiwan University Hospital, Taipei, Taiwan

**Keywords:** Parkinson’s disease, electron transport chain, mitochondria quality control, mitophagy, apoptosis

## Abstract

Parkinson’s disease (PD) is known as a mitochondrial disease. Some even regarded it specifically as a disorder of the complex I of the electron transport chain (ETC). The ETC is fundamental for mitochondrial energy production which is essential for neuronal health. In the past two decades, more than 20 PD-associated genes have been identified. Some are directly involved in mitochondrial functions, such as *PRKN, PINK1*, and *DJ-1*. While other PD-associate genes, such as *LRRK2*, *SNCA*, and *GBA1*, regulate lysosomal functions, lipid metabolism, or protein aggregation, some have been shown to indirectly affect the electron transport chain. The recent identification of *CHCHD2* and *UQCRC1* that are critical for functions of complex IV and complex III, respectively, provide direct evidence that PD is more than just a complex I disorder. Like UQCRC1 in preventing cytochrome *c* from release, functions of ETC proteins beyond oxidative phosphorylation might also contribute to the pathogenesis of PD.

## Introduction

Parkinson’s disease (PD) is the second most common neurodegenerative disease worldwide with 6.1 million patients globally in 2016 (GBD 2016 Parkinson’s Disease Collaborators, 2018), and up to 12.9 million people estimated to be affected by 2040 (Dorsey and Bloem, 2018). PD is clinically characterized by slowly progressive, levodopa-responsive bradykinesia with either rigidity or resting tremor (Postuma et al., 2015). Dopaminergic neuronal loss and Lewy body formation in the substantia nigra pars compacta are the hallmarks of most PD pathology.

Etiologies of PD are complex. Most individuals with PD are idiopathic. Only 10% of them showed a clear Mendelian inheritance (Hardy et al., 2009), and environmental factors such as pesticides, heavy metals, illicit substances, and diets also contribute (Ball et al., 2019). In 1983 four cases of levodopa-responsive parkinsonism were described after intravenous injection of synthetic heroin containing 1-Methyl-4-phenyl-1,2,3,6-tetrahydropyridine (MPTP) (Langston et al., 1983). The neurotoxicity of MPTP results from its oxidized metabolite, 1-methyl-4-phenylpyridine (MPP^+^) (Levitt et al., 1982; Glover et al., 1986), which damages oxidative phosphorylation (OXPHOS) by inhibiting the electron transport chain (ETC) of mitochondria (Nicklas et al., 1985). The keystone discovery initiated an era of mitochondrial pathology in PD. Nowadays, a plethora of evidence from electrophysiological and anatomical perspectives has shown that mitochondrial health is essential in the integrity of dopaminergic neurons, especially those in SNc (Bolam and Pissadaki, 2012; Müller et al., 2018). Therefore, to disentangle the complex pathophysiology of PD, mitochondria is undoubtedly one of the key players that should not be missed.

In this review, we will briefly introduce current knowledge about ETC and OXPHOS and their relationships with PD. We will then summarize some of the well-known and newly identified PD-associated genes and their direct or indirect influences on ETC. Finally, we discuss the OXPHOS-independent functions of ETC proteins and their possible implications in PD pathogenesis.

### Electron Transport Chain and Parkinson’s Disease

**FIGURE 1:**
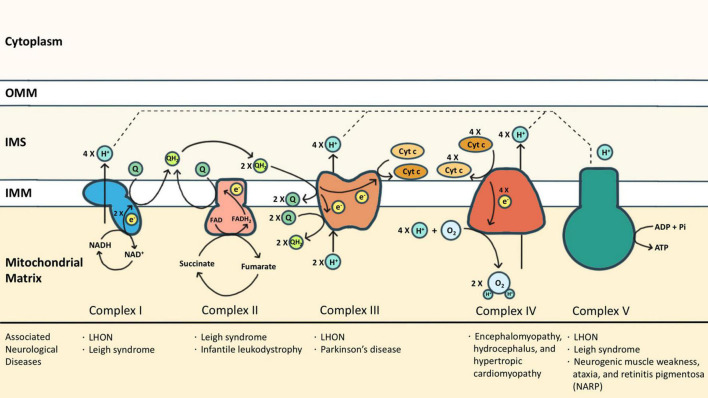
The electron transport chain (ETC) consists of complexes I (cI) to V (cV), as well as two free electron carriers, CoQ and cyt *c*. NADH and FADH_2_ donated electrons to cI and cII, respectively, causing reduction of CoQ into CoQH_2_. The CoQH_2_ is in turn oxidized by cIII where the electrons are delivered to cyt *c*. The reduced cyt *c* was then oxidized by cIV where the oxygen molecule was reduced as the terminal electron acceptor. Protons accumulated in the intermembrane space during oxidative phosphorylation via cI, cIII, and cIV, and are essential for cV to drive ATP synthesis. Some neurological diseases associated with mutations of cI-cV are listed.

Mitochondria are the powerhouses of eukaryotic cells. This double membrane-bound organelle generates most adenosine triphosphate (ATP) through OXPHOS, processed by ETC embedded in the inner mitochondrial membrane (IMM). ETC is composed of transmembrane complexes I (cI) to V (cV) and two electron carriers, the ubiquinone (i.e., CoQ) and the cytochrome *c* (cyt *c*). For ATP production, electrons are transferred from NADH and FADH_2_ to oxygen *via* the transport chain, coupled with the generation of a proton gradient across IMM (Zhao et al., 2019; Figure 1).

Complex I (cI) is an NADH-ubiquinone oxidoreductase that pumps four protons into the intermembrane space (IMS) upon each NADH oxidation. cI consists of three modules: the N, Q, and P modules. NADH generated from the tricarboxylic acid cycle delivers its electron to the N module. The electron was then passed to the Q module where the CoQ is reduced to ubiquinol (CoQH_2_) and induces conformational changes of the P module to allow proton translocation (Giachin et al., 2016). Similarly, cII also transfers electrons to CoQ, but by dehydrogenizing succinate to fumarate, which is a part of the tricarboxylic acid cycle. cII is composed of two subunits: the enzymatic subunit (SDHA-SDHB) in the matrix and the anchoring subunit (SDHC-SDHD) across the IMM. The enzymatic subunit exploits two electrons from succinate and passes them through a series of FeS clusters to the anchoring subunit where CoQ is reduced to CoQH_2_. Different from cI, no proton is translocated for the reactions that occur in cII (Rutter et al., 2010). Electrons from both cI and cII are carried by CoQH_2_ to enter the Q cycle processed by cIII. The catalytic activity of cIII depends on three redox-active subunits: cytochrome b (MT-CYB), the Rieske iron-sulfur [Fe2-S2] protein (UQCRFS1), and cytochrome c1 (CYC1) (Yang and Trumpower, 1986). Electrons donated by CoQH_2_ were transduced to cyt *c via* these three subunits sequentially. Each Q cycle generates two reduced cyt *c* and transports two protons (Crofts, 2004). The reduced *cyt c* then shuttles electrons to cIV, namely the cyt *c* oxidase (COX), whose catalytic core includes subunits 1, 2, and 3. Electrons are exploited from reduced cyt *c* at subunit 2 and reduce molecular oxygen to water at subunit 1. One proton is simultaneously translocated to IMS *via* subunit 3 upon each *cyt c* oxidization (Wilson and Prochaska, 1990). The oxidized cyt *c* then returns to its pool. In addition to working as an electron carrier, the oxidized cyt *c* also serves as a scavenger of reactive oxygen species (ROS) (Wang et al., 2003), a cardiolipin peroxidase (Kagan et al., 2005), and an apoptosis activator once released to the cytosol (Ow et al., 2008). The functions of cyt *c* beyond OXPHOS are discussed in the latter part of this review.

As the final step of OXPHOS, the proton gradient established by cI, cIII, and cIV drives cV to generate ATP. cV consists of two functional domains: F_1_ and F_0_. Protons passing through the F_0_ domain across the IMM to the matrix release the energy provided by the proton electrochemical gradient. The F_1_ domain at the matrix then uses the energy to charge cells by phosphorylating ADP to ATP (Jonckheere et al., 2012). Each ATP synthesis requires the translocation of 3 or 4 protons (Van Walraven et al., 1996; Guerra et al., 2002).

Dysfunction of ETC has been associated with various neurological diseases from infantile-onset neurodevelopmental regression (such as Leigh syndrome) to adult-onset optic neuropathy or peripheral neuropathy (such as Leber hereditary optic neuropathy (LHON) in cI deficiency, and neurogenic muscle weakness, ataxia, and retinitis pigmentosa (NARP) in cV deficiency) (Gorman et al., 2016). Neurodegeneration diseases, especially PD, are also associated with ETC dysfunction. PD has been branded as a “cI disease” because of the discovery of MPP^+^ (the metabolites of MPTP) which is a cI inhibitor (Nicklas et al., 1985; Ramsay et al., 1986). Rotenone, another commonly reported PD risk factor, is also a cI inhibitor (Betarbet et al., 2000). In post-mortem studies of PD patients, cI functional deficiency is found in substantia nigra or frontal cortex (Schapira et al., 1990; Parker et al., 2008). Similar cI deficiency is also found in non-brain tissues including platelets, skeletal muscles, or fibroblasts, indicating it is a systematic phenomenon (Parker et al., 1989; Wiedemann et al., 1999). Also, reports of cIII and cIV function in PD have been inconsistent (Mizuno et al., 1990; Wiedemann et al., 1999). Impairment of ETC causes excessive ROS, disruption of the electrochemical potential of the proton gradient, insufficient ATP production, and even cell death. Although it is widely observed that ETC functions are compromised in PD, genetic mutations of ETC proteins (either nuclear-encoded or mitochondrial-encoded) are rarely linked to PD. The discrepancy might suggest that the ETC dysfunction is secondary to other mechanisms that closely regulate mitochondria. In other words, ETC represents a hub where different PD-causing patho-mechanisms converge.

### PD-Associated Genes and the ETC

**FIGURE 2:**
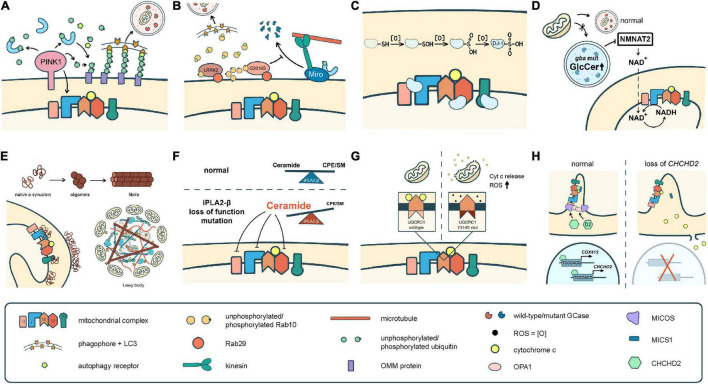
Different pathomechanisms converge on mitochondria in Parkinson’s disease. **(A)** PINK1 and parkin mediate mitochondrial quality control processes such as mitophagy. PINK1 is also required for phosphorylation of Ndufa10 to facilitate the reduction of ubiquinone by complex I. **(B)** Mutations in LRRK2 block mitophagy by preventing the degradation of Miro or by trapping Rab10 whose interaction with OPTN is pivotal for autophagy/mitophagy. **(C)** DJ-1 scavenges ROS through sequential oxidation at Cys106. The oxidized DJ-1 acts as a chaperone to facilitate the assembly and activities of cI, cIV, and cV. **(D)** Accumulation of GlcCer and other lipids in GCase mutants impairs lysosomal functions. Mutations in GCase reduces the expression of NMNAT2, resulting in a significant reduction of NAD + /NADH. **(E)** α-Syn monomers or oligomers interrupt the activities of cI and cV. The fibrilized α-Syn triggers Lewy body formation which sequestrates abundant mitochondria. **(F)** Deficits in iPLA2-β cause lipid imbalance that may interrupt ETC functions. **(G)** UQCRC1 is critical in cIII assembly and functions, and also prevents cyt *c* release. **(H)** CHCHD2 is a chaperone of cIV, activates *COX4I2* and its own expression, complexes with MICS to prevent cyt *c* release, and regulates the cristae structure by stabilizing OPA1 and MICOS complex.

To date, over 20 PD-related genes have been discovered. Although these familial PD cases are not common, and some may even have atypical features such as early onset (<50 years of age at onset) or early cognitive or psychiatric involvement, they provide great insights into the pathogenesis of PD. Many of the PD-causing genes affecting quality control systems of mitochondria, lysosomal regulation, or lipid/protein homeostasis, thus directly or indirectly influence ETC functions. In the following section, we will discuss the best-known genes and some newly identified ones with a focus on their influences on ETC (Figure 2 and Tables 1, 2).

**TABLE 1:** PD-associated genes and their influences on each ETC complex.

Gene	Mechanism	Complex I	Complex II	Complex III	Complex IV	Complex V	References
*PRKN and PINK1*	Protein turnover	+	+	+	+	+	Vincow et al., 2013
	Translation derepression	+		+		+	Gehrke et al., 2015
*PINK1*	Phosphorylation	+					Morais et al., 2014
*PARK7*	Chaperone	+			+	+	Hayashi et al., 2009; Heo et al., 2012; Chen et al., 2019
*PLA2G6*	Ceramide metabolism	+	+				Kinghorn et al., 2015
*GBA*	Unknown, probably regulating mitophagy, GlcCer metabolism, and NAD + production	+	+	+			Osellame et al., 2013; Schöndorf et al., 2018
*LRRK2*	Unknown, probably regulating mitophagy	+	+		+		Mortiboys et al., 2010
*SNCA*	Mutant monomers or oligomers	+				+	Chinta et al., 2010; Reeve et al., 2015; Ludtmann et al., 2018
	Fibril-induced Lewy body formation	+	+			+	Mahul-Mellier et al., 2020
*CHCHD2*	Chaperone				+		Aras et al., 2015; Meng et al., 2017
	Transcription factor				+		Aras et al., 2015
*UQCRC1*	cIII core subunits			+			Unni et al., 2019; Lin et al., 2020

*“ + ” indicates evidence of direct or indirect interactions but the exact involved subunits are still unclear.*

**TABLE 2:** Canonical OXPHOS functions of the mitochondrial complex subunits affected by PD-associated genes in Table 1.

Complex	Subunits*	Functions	Associated PD genes
Complex I	*NDUFB8*	An accessory subunit of the P module	*PRKN, PINK1*
	*NDUFS2*	A core subunit of the Q module	*PRKN, PINK1*
	*NDUFB11*	An accessory subunit of the P module	*PRKN, PINK1*
	*NDUFA7*	An assembly subunit	*PRKN, PINK1*
	*NDUFV2*	A core subunit of N module	*PRKN, PINK1*
	*NDUFA5*	An accessory subunit of Q module	*PRKN, PINK1*
	*NDUFA9*	An accessory subunit of Q module	*PRKN, PINK1*
	*NDUFA13*	An accessory subunit of P module	*PRKN, PINK1*
	*NDUFA8*	An accessory subunit of P module	*PRKN, PINK1*
	*NDUFB10*	An accessory subunit of P module	*PRKN, PINK1*
	*NDUFS3*	A core subunit of the Q module	*PRKN, PINK1*
	*NDUFS1*	A core subunit of the N module	*PRKN, PINK1, PARK7*
	*NDUFA10*	An accessory subunit of P module	*PINK1*
	*ND1*	An core subunit of P module	*PARK7*
Complex II	*SDHB*	An enzymatic subunit	*PRKN, PINK1*
Complex III	*UQCRB*	Maintaining Cytochrome b stability with UQCRQ	*PRKN, PINK1*
	*UQCRC2*	A core subunit of cIII; *in situ* processing of UQCRFS1 (probable)	*PRKN, PINK1*
	*UQCRC1*	A core subunit of cIII; *in situ* processing of UQCRFS1 (probable)	*-*
	*UQCRFS1*	A core subunit of cIII, responsible for transducing electrons from Cytochrome b to Cytochrome c-1	*UQCRC1*
	*CYC1*	To reduce cytochrome *c*	*UQCRC1*
	*UQCRQ*	Maintaining Cytochrome b stability with UQCRB	*UQCRC1*
	*UQCR10*	Unclear	*UQCRC1*
	*UQCR11*	Unclear	*UQCRC1*
Complex IV	*COX5A*	Response to hypoxia and regulate NO production	*PRKN, PINK1*
	*COX4I1 and-2*	Modulation of oxygen affinity	*PRKN, PINK1, CHCHD2*
	*COX2*	To oxidize cytochrome *c*	*PRKN, PINK1*
	*NDUFA4*	CIV maintenance	*PARK7*
Complex V	*ATP5L, 5L2*	A subunit of the F0 domain	*PRKN, PINK1*
	*ATP5H, ATP5HL1*	A subunit of the F0 domain	*PRKN, PINK1*
	*ATP5F1*	A subunit of the F0 domain	*PRKN, PINK1*
	*ATP5PO*	A subunit of the F0 domain	*PRKN, PINK1*
	*ATP5F1B*	A subunit of the F1 domain	*PARK7, SNCA*

*All designated by human gene names.

#### PINK1 and PRKN: Key Players of Mitochondria Quality Control

Mutations in *PRKN* (previously known as *PARK2*, which encodes parkin) and *PINK1* are the most common causes of early onset PD. *PRKN* mutants on 6q25.2-27 were found in 1998 (Kitada et al., 1998), and *PINK1* mutants on 1p35-1p36 were identified in 2001–2002 (Valente et al., 2001, 2002, 2004). Mutations in the two genes have since been reported worldwide (Kilarski et al., 2012). Early onset PD associated with either gene showed similar clinical features, including slow disease progression, good levodopa response, lower limb dystonia, early psychiatric symptoms, and a higher likelihood of motor complications than idiopathic PD (Koros et al., 2017). Pathologically, Lewy bodies can be absent in patients with either *PRKN* or *PINK1* mutations (Poulopoulos et al., 2012; Takanashi et al., 2016). These similarities reflect their common mechanism in PD pathogenesis.

It is now known that parkin, along with PINK1, is critical for mitochondria quality control through various pathways including mitophagy, formation of mitochondrial-derived vesicles, mitochondrial fission, and facilitation of mitochondrial biogenesis (Ge et al., 2020). When mitochondria are jeopardized, PINK1 is recruited to and stabilized on the OMM. The stabilized PINK1 then recruits and activates parkin, an E3 ligase that assembles ubiquitin chains on its substrates including OMM proteins which subsequently recruits ubiquitin-binding autophagy receptors and ultimately leads to autophagosome formation and lysosomal degradation. Other substrates of parkin include Mitofusin-1 and -2, whose proteolysis divert mitochondria from fusion into fission (Tanaka et al., 2010; Ziviani et al., 2010); parkin interacting substrate (PARIS, also known as ZNF746), whose degradation stimulates mitochondrial biogenesis (Shin et al., 2011; Lee et al., 2017); and also hnRNP F, a translation repressor that regulates the localized synthesis of nuclear-encoded mitochondrial proteins (including some ETC components) (Lee et al., 2017). In addition to PINK1-dependent phosphorylation, other post-translational modifications of parkin, such as S-nitrosylation, are also critical for the E3 ligase activity and solubility of parkin and have been comprehensively reviewed elsewhere (Chakraborty et al., 2017).

The integrity of ETC is highly correlated with the PINK1-Parkin-mediated quality control mechanisms. Fibroblasts or leukocytes with parkin loss-of-function mutations showed impaired cI activities (Müftüoglu et al., 2004; Mortiboys et al., 2008), and human fibroblasts or mouse embryonic fibroblasts of *PINK1* mutants also showed cI dysfunction (Morais et al., 2009; Amo et al., 2014). The turnover rates of mitochondrial proteins could be an indicator of mitochondrial quality control. A study using fly heads showed that ETC mitochondrial proteins had a greater dependence on parkin or Pink1 than other non-ETC mitochondrial proteins. Nineteen of the ETC proteins had longer half-lives in *parkin* mutants than in autophagy-impaired *Atg7* mutants, suggesting the turnover of these proteins may be achieved *via* an a selective manner. More than half belong to cI (10/19), while proteins from the other complexes are also affected (1 in cII, 2 in cIII, 3 in cIV, and 3 in cV) (Vincow et al., 2013). The predominance of cI proteins likely explains why cI dysfunction is invariably reported *in PRKN* or *PINK1* mutants.

Activities of cI could also be regulated by PINK1 independent of parkin. An accessory subunit of cI, Ndufa10, is pivotal for the ubiquinone-reducing capacity of cI, and the phosphorylation of Ndufa10 requires PINK1 (Morais et al., 2014). Overexpression of Ndufa10 or expression of its phosphomimetics could reverse the defects in pink^*B*9^-null mutant *Drosophila*, but not *parkin* mutants (Morais et al., 2014; Pogson et al., 2014). Ndufa10 phosphorylation also rescued the ROS-induced apoptosis in PINK1 knockout mouse fibroblasts (Morais et al., 2014), suggesting that PINK1 is anti-apoptotic. The anti-apoptotic effects of PINK1 are also attributed to the phosphorylation of the mitochondrial chaperone TRAP1 (also known as Hsp75) which prevents cyt *c* release from mitochondria (Pridgeon et al., 2007). In tumor cells, TRAP1, phosphorylated by ERK1/2 and Src, prohibited the generation of ROS by inhibiting cII and cIV, respectively (Masgras et al., 2017). Whether the phosphorylation of TRAP1 by PINK1 has any influence on ETC functions awaits further investigation.

#### DJ-1: A Prominent Chaperone in Mitochondria

*DJ-1* (also known as *PARK7*) mutations cause autosomal recessive, early onset PD. It was initially reported in 2003 from two genetically isolated consanguineous families (Bonifati et al., 2003), and has a frequency of about 0.4% in early onset PD, much lower than that of *PRKN* and *PINK1* (Kilarski et al., 2012).

DJ-1 is ubiquitously expressed (Mita et al., 2018) and serves a dual role as an oxidative sensor and also an antioxidant protein. Immunostaining of oxidized DJ-1 (oxDJ-1) revealed reactivity in SNc, striatum, and inferior olivary nucleus in postmortem brain of elders with Lewy body pathology. Co-localization of oxDJ-1 and α-synuclein (α-Syn) was also reported (Saito et al., 2014). The cysteine residue (C106) in its active site could be sequentially oxidized into the sulfenylated form (-SOH), sulfinated form (-SO_2_H), and sulfonic form (-SO_3_H) (Kinumi et al., 2004). The sulfinated form is the active form that not only prevents α-Syn fibrillation (Zhou et al., 2006) but also plays protective roles in promoting mitochondria fission and cell viability to rotenone (Blackinton et al., 2009). These results suggested that DJ-1 is an oxidative stress-dependent chaperone.

Since mitochondria are the major site of intracellular ROS production, it is intuitive that DJ-1 plays an important role in mitochondria physiology. The subcellular and submitochondrial fractionation of mouse brains shows that abundant endogenous DJ-1 is localized in mitochondria (Zhang et al., 2005). Depletion of DJ-1 significantly reduced the oxygen consumption rate, ATP production (Heo et al., 2012), and cI activity (Hayashi et al., 2009). Proteomics assays revealed improper cI assembly in DJ-1-deficient neuronal cells related to loss of CI-75kD (encoded by *Ndufs1*) (Heo et al., 2012). Direct interactions of DJ-1 with ND1 and with NDUFA4 were also reported (Hayashi et al., 2009). Of note, NDUFA4 is a subunit of cIV, rather than within cI as initially considered (Balsa et al., 2012). Whether cIV activities are influenced by DJ-1 remains unknown.

Recently, Chen et al. (2019) showed DJ-1 binds to the F_1_F_*O*_ ATP synthase β subunit. ATPase β subunit is crucial for maintaining the mitochondrial membrane potential through inhibition of proton leakage from the pores formed by the c-subunit of cV, thereby enhancing the ATP synthesis (Alavian et al., 2014). The ATPase β subunit was decreased in DJ-1 knockout mice, thus the ATP production, mitochondrial membrane potential, and neurite outgrowth of dopaminergic neurons were all compromised (Chen et al., 2019). The interactions of DJ-1 and ATP synthase β subunit again demonstrate its role as a mitochondrial chaperone.

#### PLA2G6: ETC Dysfunction and Lipid Imbalance

*PLA2G6*-associated neurodegeneration (PLAN) consists of a series of rare diseases: infantile neuroaxonal dystrophy (INAD), atypical neuroaxonal dystrophy (ANAD), dystonia-parkinsonism (DP), and autosomal recessive early onset parkinsonism (AREP). Among them, DP and AREP are both characterized by adult-onset as well as levodopa-responsiveness, and are not reported until 2009 and 2011, respectively (Paisan-Ruiz et al., 2009; Shi et al., 2011). Though previously under-reported, recent studies showed that *PLA2G6* might contribute to early onset PD as frequently as 0.54–1.3%, more common than *DJ-1* (Kumar et al., 2020; Zhao et al., 2020).

*PLA2G6* encodes a group VIA calcium-independent phospholipase A2β enzyme (iPLA2β), which is responsible for the selective hydrolysis of glycerophospholipids to generate fatty acids and lysophospholipids (Jenkins et al., 2006). In *PLA2G6*-knockout mice, granules containing collapsed mitochondria were observed in axons (Beck et al., 2011). In *iPLA2-VIA* (the homolog of *PLA2G6*) null *Drosophila*, aberrant mitochondria with reduced respiratory chain activities of cI and cII or ATP production were demonstrated (Kinghorn et al., 2015; Lin et al., 2018). These changes, however, are unlikely associated with changes in cardiolipin composition (Kinghorn et al., 2015), which is the main component of mitochondria inner membrane. Lin et al. showed that none of the glycerophospholipids significantly changed in *iPLA2-VIA* null *Drosophila*. Rather, they showed a decrease in ceramide phosphoethanolamines, and an increase in ceramides, dihydroceramides, and other sphingolipid intermediates (Lin et al., 2018). Some ceramides are associated with compromised respiratory chain activities, increased ROS production, impaired mitochondria membrane potential, mitophagy, and apoptosis (Kogot-Levin and Saada, 2014). By using liquid chromatography-tandem mass spectrometry (LC-MS/MS), it was revealed that subunit 2 of cIV might have allosteric interactions with C6-ceramide (Kota et al., 2012). Reduced cIV activities were found in the liver of ceramide synthase 2 deficient mice where very long acyl chain (C22-C24) ceramides were barely detectable while C16-ceramide was accumulated (Zigdon et al., 2013). In cells that have excessive levels of dihydroceramide and dihydrosphingolipids due to the lack of dihydroceramide desaturase 1, the activities of cI and cIV (especially the latter) were inhibited (Gudz et al., 1997). In these animal models, it is still not clear whether cIV activity is affected by *PLA2G6/iPLA2-VIA* and how the imbalance of lipids influences cI and cII.

#### GBA1: Lysosomal Dysfunction Linking to ETC Impairment

*GBA1* is not a traditional PARK-designated gene. Its biallelic mutations cause Gaucher’s disease, which is a lysosomal storage disease. However, increasing reports have identified *GBA1* variants as common risk factors of PD (Lwin et al., 2004; Iwaki et al., 2019). The combined odds ratio of any *GBA1* mutant in PD patients versus controls was as high as 5.43 (95% CI, 3.89 to 7.57) according to an international multi-center study, with the most common variants being N370S and L444P (Sidransky et al., 2009). PD patients with *GBA1* variants have a more aggressive motor deterioration and accelerated course of dementia than other PD patients (Stoker et al., 2020).

*GBA1* encodes β-glucocerebrosidase (GCase), a lysosomal enzyme hydrolyzing glucosylceramide (GlcCer) into glucose and ceramide. The enzyme activity of GCase decreases in mutation carriers and elder individuals (Rocha et al., 2015; Hallett et al., 2018). In contrast to the *PLA2G6* mutants that cause accumulation of ceramide, GCase deficiency results in the accumulation of GlcCer, as well as alterations of glucosylsphingosine (GlcSph), sphingosine (Sph), sphingosine-1-phosphate (S1P) in different brain regions (Muñoz et al., 2021). GCase deficiency impaired lysosomal recycling (Magalhaes et al., 2016) and lysosomes enriched with GlcCer accelerates and stabilizes soluble α-Syn oligomers, which eventually become amyloid fibrils (Mazzulli et al., 2011). GCase deficiency also causes significant mitochondrial morphological changes, decreased oxygen consumption rate, and reduced respiratory chain complex activities (Osellame et al., 2013; Schöndorf et al., 2018).

The mechanism of how a lysosomal enzyme such as GCase influences ETC is elusive. In *gba* knockout mice inhibited activities of cI and cII-III, but not cIV, were observed. Reduced co-localization of mitochondrial marker with LC3 and parkin recruitment suggested impaired mitophagy which might explain the compromised ETC activities (Osellame et al., 2013). However, the mitophagy theory unlikely explains the discrepancy between cIV and other complexes (Gegg and Schapira, 2016). CoQ, which transfers electrons from cI and cII to cIII, has been shown beneficial for mitochondrial function in fibroblasts from patients of Gaucher disease (de la Mata et al., 2015), but definite evidence showing deficits of CoQ in GCase depleted cells is lacking.

The ETC dysfunction can also result from alterations of mitochondrial metabolism. In iPSC neurons derived from PD patients carrying *GBA1* variants, cI perishment with decreased expression of nicotinamide mononucleotide adenylyltransferases2 (NMNAT2) was found. Supplementation of NAD^+^ precursor nicotinamide riboside rescued mitochondrial defects and autophagy. The lipidomic analysis of mitochondria showed no accumulation of GlcCer in mitochondria except for C_16_-GlcCer (Schöndorf et al., 2018). Where do the C_16_-GlcCer exactly locate and whether it regulates ETC functions are still unanswered.

#### LRRK2: A Master Kinase Regulating the Mitochondrial Quality Control System

Mutations in *LRRK2*, which encodes the leucine-rich repeat kinase 2 (LRRK2), are the most prevalent causes of autosomal dominant PD worldwide. *LRRK2*-associated PD (LRRK2-PD) are characterized by late-onset (>60 years), with clinical features and treatment response resembling idiopathic PD (Tan et al., 2019; Lesage et al., 2020; Zhao et al., 2020). G2019S is the most common variant in Europe and North America, accounting for 4% of familial PD and 1% of sporadic PD (Healy et al., 2008), while in Asia this variant is rare but prominent allelic heterogeneity was observed (Foo et al., 2017, 2020). One of the main features of LRRK2-PD is its incomplete penetrance which varies among each variant and even different ethnicities (Trinh et al., 2014). A polygenic risk score has been developed to predict the vulnerability of G2019S carriers, and genes involving the vacuolar functions, lysosomal functions, and endocytic pathways were included (Nalls et al., 2019). Recent studies suggested that mitochondrial DNA damage is also a potential biomarker in LRRK2-PD (Gonzalez-Hunt et al., 2020). Reduced cI activities and increased mitochondria DNA copy number were observed in fibroblasts derived from G2019S PD patients than from carriers (Delcambre et al., 2020), implying mitochondria in association with the incomplete penetrance of the G2019S variant.

LRRK2 is an enzyme with both kinase and GTPase activities (Berwick et al., 2019). The kinase activity of LRRK2 is responsible for phosphorylation of a subset of Rab small GTPase (Steger et al., 2016), which are important for exocytosis of synaptic vesicles, and endolysosomal and Golgi apparatus protein sorting (Bae and Lee, 2020). Most PD-associated LRRK2 mutations represent gain-of-function alleles: the G2019S and I2020T in the kinase domain cause hyper-phosphorylation of Rabs (West et al., 2005; Gloeckner et al., 2006), and R1441C/G/H in the Roc-COR domain increase kinase activities through disruption of the GTP hydrolysis activity (Weiss, 2008). Hyperphosphorylation of the Rabs disrupts their interaction with GTP/GDP exchange factors (GEFs), and Rab GDP dissociation inhibitors (GDIs), resulting in their inactivation and membrane-cytosol redistribution (Steger et al., 2016).

Accumulating evidence has shown the impairment of mitochondria in LRRK2 mutants. Fibroblasts from LRRK2-PD patients exhibited defects including reduced mitochondria membrane potential, decreased ATP production, and decreased activities of complexes I, II, and IV than healthy controls. Elongation of mitochondria and increased interconnectivity of mitochondria were also observed in LRRK2-PD fibroblasts (Mortiboys et al., 2010). LRRK2 is recruited to the mitochondrial outer membrane by mitochondria-anchored Rab29 (also called Rab7L1) (Gomez et al., 2019). *RAB29* is also a risk gene of PD identified in *PARK16* loci from several GWASs (Satake et al., 2009), and in addition to mitochondria Rab29 is also distributed at trans-Golgi network, lysosomes, and autophagic vesicles to regulate membrane trafficking, lysosome homeostasis, and axonal transport of autophagosomes with LRRK2 (Eguchi et al., 2018; Liu et al., 2018; Boecker et al., 2021). When gain-of-function LRRK2 mutants are anchored to mitochondria, it phosphorylates and traps Rab10 nearby (Gomez et al., 2019), causing decreased interaction of Rab10 with OPTN (optineurin), which is an autophagy receptor, therefore impairing mitophagy (Wauters et al., 2020). LRRK2 also regulates mitophagy through interaction with Miro, which anchors mitochondria to microtubules in mitochondria axonal transport (Hsieh et al., 2016). In mitophagy, PINK1/parkin-dependent phosphorylated Miro is targeted to proteasome degradation, thus “quarantining” damaged mitochondria from further transport (Wang et al., 2011). Upon CCCP treatment, Miro interacting with wild-type LRRK2 was degraded with time, while in the LRRK2 G2019S mutant, Miro showed decreased interaction with the mutant as well as resistance to degradation, delaying mitochondrial arrest and clearance in iPSC-derived neurons (Hsieh et al., 2016). In addition to mitophagy, LRRK2 is also involved in other mitochondrial quality control pathways like fusion and fission and its cytoskeleton dynamics and trafficking (Singh et al., 2019). Although evidence showing direct regulation of ETC by LRRK2 is limited, these quality control systems mentioned earlier do have profound impacts on ETCs. A recent *in vitro* study showed that PINK1-dependent phosphorylation of Ser111 of Rab8A antagonistically regulates the phosphorylation of Thr72 of Rab8A by LRRK2 (Vieweg et al., 2020), suggesting that there might be even closer crosstalk between the two master kinase.

#### SNCA: A Vicious Cycle of Proteinopathy and Mitochondriopathy

*SNCA* is the first identified gene associated with PD in 1997 (Polymeropoulos et al., 1997). While α-synuclein (α-Syn), which is encoded by *SNCA*, is one of the fundamental components in the Lewy body, patients with *SNCA* mutants (A30P, E46K, H50Q, G51D, A53E, and A53T) or multiplications are really rare, characterized by autosomal dominant inheritance, widely distributed age of onset (from 19 to 81 years), and more rapid cognitive impairment (Rosborough et al., 2017). Physiologically α-Syn is soluble in the cytosol, mainly located at presynaptic terminals to regulate the genesis, maintenance, and release of presynaptic vesicles (Kahle et al., 2000; Cabin et al., 2002; Burré et al., 2010). The α-synucleinopathy results from the imbalance among synthesis, aggregation, and clearance of α-Syn. As a result, aggregation of α-Syn takes place in the cytosol or around membranes. The conformations of the aggregates include oligomers, protofibrils, and fibrils (Lashuel et al., 2013). Accelerated oligomerization is the common trait among different disease mutants (Conway et al., 2000), and a higher propensity of fibrilization might correlate to the earlier onset age in A53T than A30P or E46K (Conway et al., 1998; de Oliveira and Silva, 2019). Post-translational modifications like phosphorylation at Ser129 and truncation at the carboxyl terminal are also pathogenic (Sato et al., 2011; Sorrentino and Giasson, 2020), while Ser87 is protective (Oueslati et al., 2012).

Both oligomers and fibrils (especially the amyloid fibrils) are toxic to mitochondria (Lashuel et al., 2013). Overexpression of α-Syn, cytosolic acidification, and Ser129 phosphorylated pathogenic α-Syn could all induce translocation of α-Syn to mitochondria (Cole et al., 2008; Shavali et al., 2008; Wang et al., 2019). By using a split-GFP tool, the translocated α-Syn was found distributed at the OMM and IMS but not in the matrix. Mutants (A30P and A53T) were more likely translocated to the IMS than the wild-type, and more α-Syn would be translocated to IMS upon oxidative stress or cI inhibition (Vicario et al., 2019). In turn, α-Syn inhibits cI activities in either monomer or oligomerized form (Chinta et al., 2010; Reeve et al., 2015; Ludtmann et al., 2018). Oligomeric α-Syn also selectively oxidized the ATP synthase β subunit and caused mitochondrial lipid peroxidation and increased mitochondrial permeability transition pore opening (Ludtmann et al., 2018). These suggest a vicious feed-forward loop between α-Syn aggregation and mitochondrial dysfunction.

Synergistic with oligomers, α-Syn fibrils impairs mitochondria by sequestering mitochondria and other organelles into Lewy bodies. Mahul-Mellier et al. (2020) added preformed fibrils (PFF) of α-Syn exogenously to the cultured neurons and found that at day 14 inclusions composed of loose fibrils start to interact with organelles, and at day 21 high enrichment of proteins from the mitochondria (including complexes I, II, and V), the endoplasmic reticulum, and the Golgi were detected in the Lewy body-like inclusions, corresponding to the reduction of the maximum OXPHOS capacity and maximum electron transport capacity at day 21. The important discovery links the two competitive but not conflicting theories of proteinopathy and mitochondriopathy in PD pathogenesis.

#### UQCRC1 and CHCHD2: Two Recently Found PD-Causing Genes Implicating Apoptotic Neuronal Death

The association between *CHCHD2* and autosomal dominant late-onset PD was discovered in four independent families through genome-wide linkage analysis (Funayama et al., 2015). CHCHD2 is a member of the coiled-coil-helix-coiled-coil-helix (CHCH) domain-containing protein family known to participate in mitochondrial respiration, redox regulation, membrane ultrastructure, and dynamics in the IMS (Modjtahedi et al., 2016). CHCHD2 binds with cIV and regulates its activity. Under hypoxia, CHCHD2 activates the transcription of itself and *COX4I2*, which encodes cIV subunit-4 isoform 2 (COX IV-2) (Aras et al., 2015). The findings are compatible with the clinical data showing that fibroblasts derived from a patient with homozygous A71P mutant in *CHCHD2* show reduced cIV activity in addition to cI deficiency (Lee et al., 2018). CHCHD2 is also important in maintaining the cristae structure and preventing apoptosis. Cristae are the home of ETC complexes and changes in the structure would alter the distribution of mitochondrial complexes, thus influencing the efficiency of OXPHOS (Gilkerson et al., 2003; Wilkens et al., 2013). In the flight muscles of *CHCHD2* null *Drosophila*, the mitochondrial cristae structure was disorganized (Meng et al., 2017). Cristae integrity is regulated by OPA1 and the MICOS complex (Baker et al., 2019). In cells carrying PD-linked CHCHD2 mutations, the expression level of MICOS components, such as Mitofilin, MINOS1, CHCHD3, CHCHD6, were all reduced (Zhou et al., 2019). In Drosophila, *Chchd2* knockout increases the degradation of OPA1 by peptidase YME1L (Liu W. et al., 2020). Similarly, OPA1 degradation is also observed in CHCHD2 and CHCHD10 double-knockout mice (Liu Y. T. et al., 2020). Mass spectrometry analysis reveals that CHCHD2 interacts with MICS1 (Meng et al., 2017), which is involved in cristae organization and cyt *c* stabilization, thereby prohibiting cells from apoptosis (Oka et al., 2008).

*UQCRC1* is a newly identified PD-causing gene that is clinically characterized by autosomal dominant late-onset parkinsonism with polyneuropathy (Lin et al., 2019, 2020). *UQCRC1* encodes the mitochondrial ubiquinol-cyt *c* reductase core protein 1 (UQCRC1), which is a core subunit of cIII. UQCRC1 complexes with UQCRC2 and interacts with UQCRFS1, CYC1, and other cIII subunits to regulate cIII activity (Sunitha et al., 2016; Unni et al., 2019). UQCRC1 and UQCRC2 are homologs to the mitochondrial-processing peptidase subunits and are predicted to process UQCRFS1 *in situ* during cIII assembly (Fernandez-Vizarra and Zeviani, 2018). However, *in vivo* experimental validation is required.

To date, only three *UQCRC1* human mutations have been identified and functionally validated: Y314S, I311L, and p.Ala25Glyfs*27. All three mutants show reduced maximal oxygen consumption rate, decreased ATP production, and increased ROS in SH-SY5Y cell lines. Drosophila and mice with the Y314S variant both showed degeneration of dopaminergic neurons and locomotor defects (Lin et al., 2020), providing direct evidence for the involvement of cIII in PD pathogenesis.

In addition to regulating cIII activities, UQCRC1 is also anti-apoptotic. In cardiac cells, UQCRC1 prevents ischemia/reperfusion injury by activating the PI3K/Akt/GSK-3β pathway, upregulating the anti-apoptotic protein Bcl-2, and downregulating the pro-apoptotic protein Bax (Yi et al., 2020). In *Drosophila*, our recent data reveal that uqcr-c1 associates with cyt *c* in mitochondria to gate its release (Hung et al., 2021). Previous reports have shown that complex I and II mediate signals for apoptosis (Lemarie and Grimm, 2011). Together, these findings suggest that the respiratory chain complexes are important regulators of apoptosis.

## The Many Faces of ETC Proteins: Functions Beyond Bioenergetics and Possible Implications in Neurodegeneration

Abundant studies have focused on mitochondrial quality control, lysosomal functions, apoptosis, and α-Syn aggregation in PD pathogenesis and their influences on ETC functions. In contrast, whether ETC proteins have any direct involvement in these pathways other than bioenergetics remains unclear in neurodegenerative diseases.

The roles of ETC proteins in apoptosis have been extensively studied in cancer cell biology. Both cI and cII are implicated as apoptotic sensors *via* different mechanisms. In cI, cleavage of NDUFS1 by caspase-3 inhibits cI activities, causing ROS production and collapse of mitochondria membrane potential. The cleavage-resistant mutant NDUFS1 D255A decreases ROS formation and delays the loss of plasma membrane integrity (Ricci et al., 2004), suggesting cI inhibition as an accelerator of apoptosis. A similar mechanism is found in granzyme A-induced apoptosis, in which NDUFS3 was cleaved (Martinvalet et al., 2005). For cII, the acidification of matrix in response to mitochondrial stress causes the disintegration of cII by dissociating the anchoring subunit SDHC-SDHD from the enzyme subunit SDHA-SDHB. As the SDHA-SDHB subunit is still enzymatically active, it produces excessive ROS and causes apoptosis. cII, therefore, is a pH sensor of the matrix in programmed cell death.

The roles of complexes III and IV in apoptosis are less clear, but the electron carrier between the two complexes, cyt *c*, has been known as a key player by activating apoptosis protease activating factor-1 (Apaf-1) when released to the cytosol. Post-translational modifications, especially phosphorylation, of cyt *c* have regulatory roles in both electron transport and apoptosis (Kalpage et al., 2019a). Tissue-specific phosphorylation of cyt *c* at Ser47 and Tyr97 that are enriched in porcine brain tissues and insulin-treated porcine tissues, respectively (Sanderson et al., 2013; Kalpage et al., 2019b), are both anti-apoptotic not only by inhibiting caspase-3 activities (Kalpage et al., 2019b) but also by reducing the cyt *c*-COX interactions to lower the COX reaction rate and ROS generation (Lee et al., 2006; Guerra-Castellano et al., 2018; Kalpage et al., 2019b). It is not known whether the post-translational modifications of cyt *c* alter any interactions with cIII. From the lessons of UQCRC1, we now know that cIII can keep cyt *c* from being released from mitochondria.

Emerging evidence disclosed that there are other moonlight functions of cyt *c*. Abundant cyt *c* was shown to translocate to the nucleus prior to caspase activation in the cytosol during the early phase of apoptosis (Nolin et al., 2016). The translocated cyt *c* hijacks histone chaperones such as suppressor of variegation, enhancer of zeste and trithorax (SET)/template-activating factor (TAF)-Iβ, inhibiting the nucleosome assembly/disassembly activity and DNA repairing (González-Arzola et al., 2015; Díaz-Moreno et al., 2018). DNA damage is common in many neurodegenerative disorders, including PD (Ainslie et al., 2021). Cyt *c* has also been identified as one of the components of brainstem Lewy bodies (Wakabayashi et al., 2007) and might play as a trigger in α-Syn aggregation (Hashimoto et al., 1999). α-Syn fibrils rather than mutant oligomers or monomers specifically interact with cyt *c* according to an *in vitro* study (Leitão et al., 2021). Whether similar aggregations could be replicated *in vivo* and whether post-translational modifications of cyt *c* as mentioned earlier have any effects on these moonlight functions are still unknown.

Some evidence showed that ETC proteins are also involved in mitophagy or autophagy. For example, autophagy is inhibited by the cIII inhibitors Antimycin A or myxothiazol (Ma et al., 2011). Another example is ECSIT (evolutionarily conserved signaling intermediate in Toll pathways), which is essential in cI formation (Vogel et al., 2007), found as a parkin substrate and co-localized with LC3B in CCCP-treated macrophages (Carneiro et al., 2018). Upon loss of ECSIT, parkin still accumulates on the mitochondria, but the recruitment of LC3BII to mitochondria is compromised. This suggested that parkin-induced ECSIT ubiquitination is upstream of LC3BII recruitment for the initiation of mitophagy (Carneiro et al., 2018), It is not clear whether ECSIT participates in receptor-mediated mitophagy like other mitochondrial outer membrane proteins such as FUNDC1, NIX, and BNIP3. Further investigation is needed to elucidate whether ECSIT mediates mitochondria clearance *via* a similar mechanism in the nervous system.

## Discussion

In this review, we focus on the central role of ETC in PD pathogenesis. We have discussed that ATP production by mitochondria is directly or indirectly affected by reduced turnover and altered post-translational modifications of ETC proteins, impaired assembly or instability of mitochondrial complexes, abnormal protein aggregation, and dysregulated lipid metabolism. While mutations of ETC proteins are rarely linked as a direct cause of PD (except for UQCRC1), impaired ETC proteins may not only lead to excessive ROS production, but also cause impeded mitophagy, α-Syn aggregation, and apoptosis in PD pathogenesis.

It might be argued that for the long time scale of PD progression, apoptosis is an acute cellular event that on the surface does not perfectly explain the development of PD, but when patients start to have detectable parkinsonism symptoms, there is usually at least 40–60% dopaminergic neuronal loss in the substantia nigra (Giguère et al., 2018), suggesting progressive accumulation of neuronal loss. At the organismal level, apoptosis of dopaminergic neurons occurs over time, responding to stress such as aggregated proteins or ETC dysfunction. At the cellular level, it takes time for a cell to reach the threshold that breaks the balance between pro-apoptotic and anti-apoptotic pathways. For example, ROS accumulation, alterations of mitochondrial outer membrane permeability, and the attenuation of antiapoptotic signals may fluctuate, and mutant cells with the propensity of releasing cyt *c* do not mean that it releases cyt *c* and activate caspases immediately. This concept of ‘apoptotic threshold’ has been well illustrated by a beautiful mathematical model (Rehm et al., 2006). In fact, apoptosis has been implicated in many neurodegenerative diseases, including PD (Moujalled et al., 2021). Although it remains unclear whether loss of ETC function leads to protein aggregation *in vivo*, apoptosis represents an important pathogenic mechanism that warrants our attention.

In terms of mitochondrial dysfunction, the examples of UQCRC1 and CHCHD2 have suggested that PD is not just a cI disorder. Various genetic models of PD have now shown deficits in cII to cV. The conventional belief of PD as a cI disorder might be the result of publication bias or study limitations in earlier studies. It has been shown that even in cI, the significance of the deficiency could be influenced by the purity of the samples and the assays we use (Parker et al., 2008). By using high-resolution quantitative fluorescence immunohistochemistry, Reeve *et al.* found that both cI and cIV were deficient in the remaining dopaminergic synapses of idiopathic PD (Reeve et al., 2018). By using imaging mass cytometry, a recent study revealed that there is even more widespread deficiency from complexes I to V in the postmortem human midbrain (Chen et al., 2021), contrary to the findings of earlier studies that were based on the measurements of biochemical activities only.

Mitochondrial therapies have been proposed as a potential treatment of PD (Thomas and Beal, 2010); however, many attempts have failed. The anti-diabetic drugs glitazones which improves mitochondrial functions (such as activities of cI and cIV) and biogenesis (Bogacka et al., 2005; Ghosh et al., 2007) did lower the incidence of PD in retrospective cohort studies (Brauer et al., 2015; Brakedal et al., 2017), but failed to show benefits in modifying disease progression of early PD (NINDS Exploratory Trials in Parkinson Disease (NET-PD) FS-ZONE Investigators, 2015). The randomized clinical trial of high dose CoQ did not slow disease progression (Beal et al., 2014). The limited bioavailability of CoQ has been considered as one of the main reasons for its failure (Bhagavan and Chopra, 2007). Longer follow-up or earlier administration of the drugs at prodromal stages may be necessary to reveal the potential benefits. Idebenone, a synthetic short-chain analog of CoQ with improved lipophilicity and bioavailability (Becker et al., 2010), having been approved as an orphan drug in treating LHON by European Medicines Agency (EMA) (European Medicines Agency [EMA], 2021) and Duchenne muscular dystrophy by the United States Food and Drug Administration (FDA) (Pharmaceuticals, 2015), is tested in two PD clinical trials: (1) Idebenone Treatment of Early Parkinson’s Disease symptoms (ITEP) (NCT03727295), and (2) A Study of Efficacy and Safety of Idebenone vs. Placebo in Prodromal Parkinson Disease (SEASEiPPD) (NCT04152655). The recruitment status of the former is due to end in May 2021, while the latter is still recruiting and is scheduled to complete by January 2023.

In conclusion, ETC dysfunction is a common feature in PD, secondary to various pathomechanisms. Increasing evidence has shown that cI is not the only affected ETC complex. Other complexes, such as cIII and cIV, are also involved. ETC dysfunction not only reduces ATP production but also induces apoptosis or impaired mitophagy that undermines neuronal viability in PD. So far there is still no silver bullet to prevent or to ameliorate PD progression. Non-etheless, new compounds or existing drugs await testing as potential therapeutic strategies for PD *via* improving ETC functions (Singh et al., 2021).

## Author Contributions

J-LL, T-YL, and C-CC: conceptualization. J-LL and T-YL: writing – original draft. C-CC: funding acquisition and supervision. All authors writing – review and editing.

## Conflict of Interest

The authors declare that the research was conducted in the absence of any commercial or financial relationships that could be construed as a potential conflict of interest.

## Publisher’s Note

All claims expressed in this article are solely those of the authors and do not necessarily represent those of their affiliated organizations, or those of the publisher, the editors and the reviewers. Any product that may be evaluated in this article, or claim that may be made by its manufacturer, is not guaranteed or endorsed by the publisher.
